# Urokinase-type plasminogen activator (uPA) and its receptor (uPAR) promote neurorepair in the ischemic brain

**Published:** 2017-06-06

**Authors:** Paola Merino, Ariel Diaz, Manuel Yepes

**Affiliations:** 1 Department of Neurology and Center for Neurodegenerative Disease, Emory University School of Medicine, Atlanta, Georgia 30322, USA; 2 Division of Neurosciences, Yerkes National Primate Research Center, Atlanta, Georgia 30329, USA; 3 Department of Neurology, Veterans Affairs Medical Center, Atlanta, Georgia 30033, USA

**Keywords:** Urokinase-type plasminogen activator (uPA), urokinase-type plasminogen activator receptor (uPAR), plasmin, cerebral ischemia, neurorepair, low density lipoprotein receptor-related protein 1 (LRP1)

## Abstract

Despite the fact that ischemic stroke has been considered a leading cause of mortality in the world, recent advances in our understanding of the pathophysiological mechanisms underlying the ischemic injury and the treatment of acute ischemic stroke patients have led to a sharp decrease in the number of stroke deaths. However, this decrease in stroke mortality has also led to an increase in the number of patients that survive the acute ischemic injury with different degrees of disability. Unfortunately, to this date we do not have an effective therapeutic strategy to promote neurological recovery in these growing population of stroke survivors. Cerebral ischemia not only causes the destruction of a large number of axons and synapses but also activates endogenous mechanisms that promote the recovery of those neurons that survive its harmful effects. Here we review experimental evidence indicating that one of these mechanisms of repair is the binding of the serine proteinase urokinase-type plasminogen activator (uPA) to its receptor (uPAR) in the growth cones of injured axons. Indeed, the binding of uPA to uPAR in the periphery of growth cones of injured axons induces the recruitment of β1-integrin to the plasma membrane, β1-integrin-mediated activation of the small Rho GTPase Rac1, and Rac1-induced axonal regeneration. Furthermore, we found that this process is modulated by the low density lipoprotein receptor-related protein (LRP1). The data reviewed here indicate that the uPA-uPAR-LRP1 system is a potential target for the development of therapeutic strategies to promote neurological recovery in acute ischemic stroke patients.

## Introduction

Ischemic stroke is the fifth cause of death in USA and a leading cause of morbidity and disability in the world ^[1]^. Accordingly, the World Health Organization estimates that each year 15 million people worldwide and 795 000 in USA suffer stroke. Remarkably, the successful development of new therapeutic approaches to treat acute ischemic stroke patients, including thrombolysis with tissue plasminogen activator (tPA) ^[2]^ and mechanical removal of the occluding clot ^[3]^, has led not only to a 33.7% and 18.2 % drop in the relative rate of stroke death and the actual number of stroke deaths, respectively ^[1]^, but also to an increase in the number of patients that survive an acute ischemic stroke with different degrees of neurological disability. Unfortunately, to this date there is no effective therapeutic strategy to promote neurological recovery in this growing population of stroke survivors.

The degree of functional disability following an ischemic stroke depends to a great extent on the capacity of neurons to withstand and recover from the harmful effects of the ischemic insult. Indeed, it is estimated that one minute of cerebral ischemia is enough to destroy 1.9 million neurons, 14 billion synapses and 7.5 miles of myelinated axons ^[4]^. Despite these appalling effects on neuronal integrity and function, the ischemic injury also activates endogenous mechanisms that promote the recovery of those neurons that have survived the acute stages of the ischemic injury. Our recent work indicates that one of these mechanisms is the binding of the serine proteinase urokinase-type plasminogen activator (uPA) to its receptor (uPAR). These findings are of significant importance because they indicate that the uPA-uPAR system is a potential target for the development of effective therapeutic strategies to promote neurological recovery in the rapidly growing population of ischemic stroke survivors. Furthermore, these observations have a high translational relevance because it is well known that in contrast with the peripheral nervous system, the central nervous system has a limited capacity for regeneration. Therefore, the discovery of a potential therapeutic target to promote neurological recovery may have a significant impact on the quality of life and productivity of a large number of ischemic stroke patients.

## The uPA-uPAR system

uPA is assembled by an amino terminal epidermal growth factor (EGF)-like domain that contains the uPAR binding residues, a kringle domain and a carboxy-terminal proteolytic domain ^[5]^. The first two regions form the amino terminal fragment (ATF), which contains the interaction site with the receptor but lacks the enzymatic activity. As it will be discussed below, we found that ATF promotes axonal regeneration via activation of the Rho family small GTPase Rac1, thus indicating that uPA induces neurorepair by plasminogen-independent activation of an intracellular cell signaling pathway ^[6]^. Besides its biological significance, this finding has important translational implications because it indicates that the potential administration of uPA’s ATF to promote neurological recovery in ischemic stroke patients is devoid of plasmin-induced complications, namely intracerebral hemorrhage.

uPAR is an extracellular receptor assembled by three globular domains anchored to the membrane through a glycosylphosphatidylinositol (GPI) tail ^[7]^. uPAR not only interacts with pro-uPA, uPA, uPA’s ATF, and the extracellular matrix (ECM) protein vitronectin (VN) ^[8]^, but also can be released from the plasma membrane by the cleavage of either its GPI tail or different sites of its globular domains to produce a soluble form (suPAR) ^[9]^. It has been demonstrated that uPAR modulates cell adhesion, differentiation, apoptosis, proliferation and migration not only through its proteolytic function but also by activating intracellular cell signaling pathways ^[10–12]^. These observations will be discussed below and are in line with our findings indicating that the binding of either recombinant uPA (ruPA) or endogenous uPA to uPAR promotes axonal recovery in the ischemic brain. Importantly, because uPAR is bound to the plasma membrane by a GPI tail, it requires transmembrane co-receptors to activate intracellular cell signaling pathways ^[8]^. As it also will be discussed below, we found that two co-receptors, namely β1-integrin and the low-density lipoprotein receptor-related protein (LRP1), mediate the effect of uPA-uPAR binding on axonal recovery.

## uPA-uPAR expression in the central nervoussystem

The expression of uPA and uPAR in the developing central nervous system (CNS) is particularly high in neurons ^[6, 13, 14]^, microglia ^[15]^ and astrocytes ^[16]^. However, during maturation it progressively decreases to reach very low levels in the adult brain. During development uPA-uPAR binding promotes neuritogenesis and neuronal migration via a combination of proteolytic and non-proteolytic mechanisms ^[17, 18]^. More specifically, during the early stages of development uPAR regulates the reorganization of the cytoskeleton in post-mitotic neurons via activation of integrins and the focal kinase adhesion (FAK) pathway ^[17]^, thus promoting axonal growth, and neuronal migration ^[19]^ and branching ^[20]^. Interestingly, it has also been reported that uPAR participates in the formation of those neuronal circuits that underlie language and cognition, and that dysregulation of the uPA-uPAR signaling pathway is related with the development of epilepsy ^[21]^. As stated above, the expression of neuronal uPAR varies according to the developmental stage. Hence, while in DIV 3 neurons uPAR is abundantly found in the cell body and neurites, at DIV 7 is mainly detected in the axon shaft and growth cones, and at DIV 16 its expression is restricted to the distal segment of some axons and very few growth cones ^[6]^. Remarkably, following axonal injury the expression of uPAR in adult neurons increases again, particularly in growth cones of injured axons, to levels comparable to those observed during the early stages of development ^[6]^. The expression of uPA in the adult brain follows a pattern very similar to uPAR: low in the healthy brain and high following an injury ^[13]^. In summary, the experimental evidence available to this date indicates that the expression of neuronal uPA and uPAR is high in the early stages of development and decreases to almost undetectable levels in mature cells. However, the expression of both, the ligand and its receptor, increases in neurons following different forms of injury. These findings are in line with observations by others indicating that the expression of uPAR increases within the first few hours of peripheral nerve ^[22]^, spinal cord ^[23]^ and cortical neurons ^[6, 13]^ injury, and has led to propose that uPAR is a marker of central nervous system damage and a potential therapeutic target to promote neurorepair.

## uPA and uPAR in the ischemic brain

Because cerebral ischemia is one of the most frequent causes of brain injury ^[24]^, then to study the role of uPA and uPAR in the injured brain we used an *in vitro* model of hypoxia in which neurons are exposed to oxygen and glucose deprivation (OGD) conditions, and an *in vivo* model of cerebral ischemia in which the middle cerebral artery is occluded (MCAO) with a suture during different periods of time. First we measured the release of uPA from wild-type (Wt) adult cerebral cortical neurons exposed to 60 minutes of OGD. Unexpectedly, we found that neurons do not release uPA while they are exposed to OGD but 6 – 24 hours after they begin recovering from the hypoxic injury ^[13]^. To characterize the *in vivo* significance of these findings we quantified the expression of uPA in the ischemic tissue of Wt mice immediately after 30 and 60 minutes of MCAO, and 1 – 24 hours after 60 minutes of MCAO and successful reperfusion (recovery). As observed with neuronal cultures, we failed to detect an increase in uPA expression in the ischemic tissue during the acute phase of the ischemic injury. However, we detected a progressive increase in the concentration of uPA 3 – 24 hours later.

These observations, and studies by others proposing that uPAR as a predictor of ischemic stroke ^[25]^, led to postulate that the expression of uPA and uPAR in the ischemic brain may underlie the development of the pathophysiological processes that lead to ischemic cell death. Surprisingly, this hypothesis was proven wrong as we failed to detect a difference in the volume of the ischemic lesion between mice genetically deficient in uPA (uPA^−/−^) and their Wt littermate controls 24 hours after 60 minutes of MCAO. In contrast, we found that compared to Wt littermate controls, uPA^−/−^ and uPAR^−/−^ mice have a protracted recovery in neurological function following MCAO, and that treatment with ruPA or the release of endogenous uPA induces recovery in Wt and uPA^−/−^, but not in uPAR^−/−^ mice ^[6, 13]^. In summary, these data indicate that the expression of uPA and uPAR increase in the sub-acute, recovery stages of ischemic stroke, and suggest that uPA binding to uPAR plays a central role in the process of neurorepair following an acute ischemic injury. These observations are supported by reports from other groups indicating that uPAR modulates peripheral nerve regeneration after a crush nerve ^[21]^, and that genetic deficiency of uPA aggravates the motor deficit and increases neuronal death in an animal model of traumatic brain injury ^[26]^.

## uPAR and the axon growth cone

**Figure 1:**
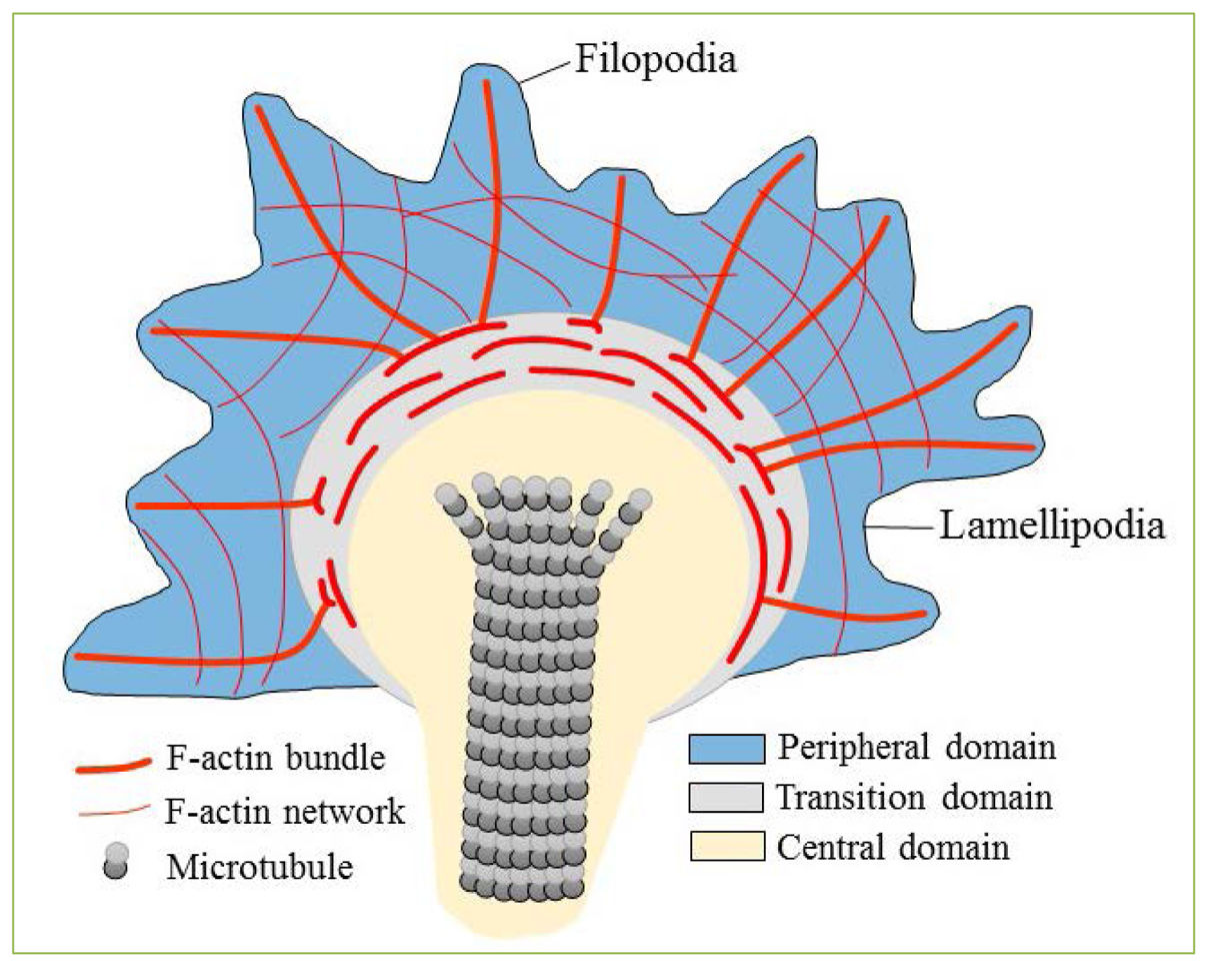
Structure of a growth cone Schematic representation depicting three well-defined areas in a growth cone: a. peripheral domain (blue) with F-actin bundles (red) that form filopodia and lamellipodia. b. central domain (gray) with microtubules that enter the growth cone from the axon shaft (yellow); and c. transition domain (gray) with contractile structures.

Axons are particularly vulnerable to CNS injury and our data indicate that the expression of uPAR increases in the growth cone of injured adult axons, and that uPA binding to uPAR improves neurological function following an ischemic injury ^[6, 13]^. Thus, based on these data we postulated that uPA binding to uPAR induces axonal recovery via a direct effect on the growth cone. The formation of growth cones plays a pivotal role not only during development but also in the initial stages of axonal regeneration. Indeed, the recovery and regeneration of an injured axon requires the establishment of a polarized extension guided by a newly formed growth cone that harbors three well-defined areas (Figure 1): a) a peripheral domain with F-actin bundles that form filopodia and lamellipodia; b) a central domain with microtubules that enter the growth cone from the axon shaft; and c) a transition area between the peripheral and central domains that contains actomyosin contractile structures ^[27]^. To study the effect of uPA binding to uPAR on the growth cones of injured axons, we developed an *in vitro* system in which a wound injury is performed on a mantle of axons that radially grows from neurosphere-like aggregates (NLA) prepared from cerebral cortical neurons ^[6]^. With this model we found that the axonal injury is followed by a rapid increase in the expression of uPAR in the filopodia of newly formed growth cones, and that the binding to uPAR of either ruPA [administered at the same doses used to treat acute ischemic stroke patients ^[28]^], or endogenous uPA, accelerates the regrowth of new axons from these growth cones ^[6]^.

## uPA-uPAR binding promote axonal recovery in the ischemic brain

As stated above, myelinated axons are particularly sensitive to the deleterious effects of cerebral ischemia. Thus, to investigate whether uPA-uPAR binding also promotes axonal recovery *in vivo* in the ischemic brain, we used an animal model in which the stereotaxic injection of endothelin-1 induces a well-defined area of ischemia in the internal capsule (IC), a subcortical structure formed by bundles of axons of pyramidal neurons that project from the cerebral cortex and other structures to the spinal cord, and that is frequently affected in ischemic stroke patients. Using this experimental design we found that cerebral ischemia increases the expression of axonal uPAR *in vivo* and that the binding of either ruPA or endogenous uPA to uPAR promotes axonal recovery and functional improvement.

## Membrane recruitment of β1-integrin mediates uPA/uPAR-induced neurorepair

As stated above, uPAR is a GPI-anchored protein that needs transmembrane co-receptors to activate intracellular cell signaling pathways. A first clue to identify the co-receptor that mediates the observed effect of uPA/uPAR on neurorepair was provided by the finding that uPA induces axonal regeneration *in vitro* in the presence of fibronectin, but not vitronectin, laminin or collagen ^[6]^. This finding indicates that the interaction between a co-receptor, most likely an integrin, and the extracellular matrix (ECM) is required for uPA/uPAR to induce axonal recovery.

Integrins are a family of (α-β) heterodimeric receptors that mediate both cell-cell and cell-matrix interactions in a wide variety of cell types ^[29]^. In the brain, integrins have been studied during development, where they participate in neuroblasts migration ^[30]^ and axonal and dendritic outgrowth, via their ability to interact with the ECM ^[31]^. An interaction between uPAR and different integrin subunits, mostly β1, β3 and β6, has been described by biochemical and computational technics ^[32–35]^. Because the β1 integrin subunit is the receptor for fibronectin ^[36–38]^, and since fibronectin is required in our system for uPA/uPAR to promote axonal regeneration, then we postulated that β1 integrin was the co-receptor that mediates the effect of uPA/uPAR on neurorepair. Our hypothesis was further supported by reports from other groups indicating that neuronal β1 integrin expression increases after ischemic stroke ^[39]^ and that β1 integrin promotes the regeneration of sensorial axons at long distances in the spinal cord ^[40]^. In line with our hypothesis and these observations, we found that treatment with uPA induces the recruitment of β1 integrin to the plasma membrane of cerebral cortical neurons, and that co-treatment with β1 integrin neutralizing antibodies blocks the effect of uPA-uPAR binding on axonal repair. Together, these data indicate that the interaction between β1 integrin and fibronectin mediates uPA-induced neurorepair.

## The low-density lipoprotein receptor associated protein-1 (LRP1) modulates uPA-uPAR- β1-integrin-mediated axonal repair

LRP1 is a member of the LDL receptor gene family assembled by a 515 kDa heavy chain non-covalently bound to an 85 kDa light chain containing a transmembrane and a cytoplasmic domains ^[41]^, that has been implicated not only in the internalization of multiple ligands but also in the activation of cellular signal transduction pathways. A growing body of experimental evidence indicates that LRP1 plays a pivotal role in neurotransmission, synaptic plasticity and neurite outgrowth ^[42]^. Furthermore, it has been demonstrated that LRP1 promotes axonal regeneration following peripheral nerve injury ^[43]^. Our data indicate that, as described for uPAR, following a mechanical injury LRP1 expression is also up-regulated in the filopodia of newly formed growth cones ^[6]^.

Based on these observations and the fact that LRP1 is an endocytic receptor for the complex assembled by plasminogen activator inhibitor-1, uPA and uPAR ^[44]^, then we postulated that treatment with the receptor associated protein (RAP), an endoplasmic reticulum resident chaperone that prevents the interaction between LRP1 and its ligands ^[45–47]^, would potentiate the beneficial effect of uPA on axonal recovery by preventing uPAR endocytosis. Surprisingly, our data proved that our hypothesis was incorrect and, instead, that treatment with RAP prevents uPA-induced recruitment of β1-integrin to the neuronal membrane and uPA/uPAR-induced axonal regeneration. Together, these results indicate that LRP1 does not act as an endocytic receptor in our model of axonal regeneration, but instead that it is a signaling receptor that promotes axonal repair.

## Rac-1 mediates the effect of uPA to uPAR binding on axonal regeneration

Axonal regeneration requires the reorganization of the actin cytoskeleton in the periphery of newly formed growth cones where we detected an increase in uPAR and LRP1 expression following an axonal injury ^[6]^. Because the small Rho family GTPases are known regulators of cytoskeletal rearrangement, and since RhoA and Rac1 mediate uPA-directed cell migration, then we postulated that either RhoA or Rac1, or both, mediate the effect of uPA on axonal repair. To test this hypothesis we quantified axonal regeneration in the presence of uPA and either Rac1 or RhoA inhibitors. We found that uPA activates Rac1 in neurons, and that this effect is abrogated by treatment with RAP. Moreover, Rac1 but not RhoA inhibition prevents the effect of uPA on axonal regeneration. Together, these data indicate that binding of uPA to uPAR promotes LRP1-mediated Rac1 activation and Rac1-mediated axonal regeneration. Furthermore, it confirmed our hypothesis that LRP1 acts as a signaling receptor that promotes axonal regeneration by inducing Rac1 activation.

## Conclusion

**Figure 2:**
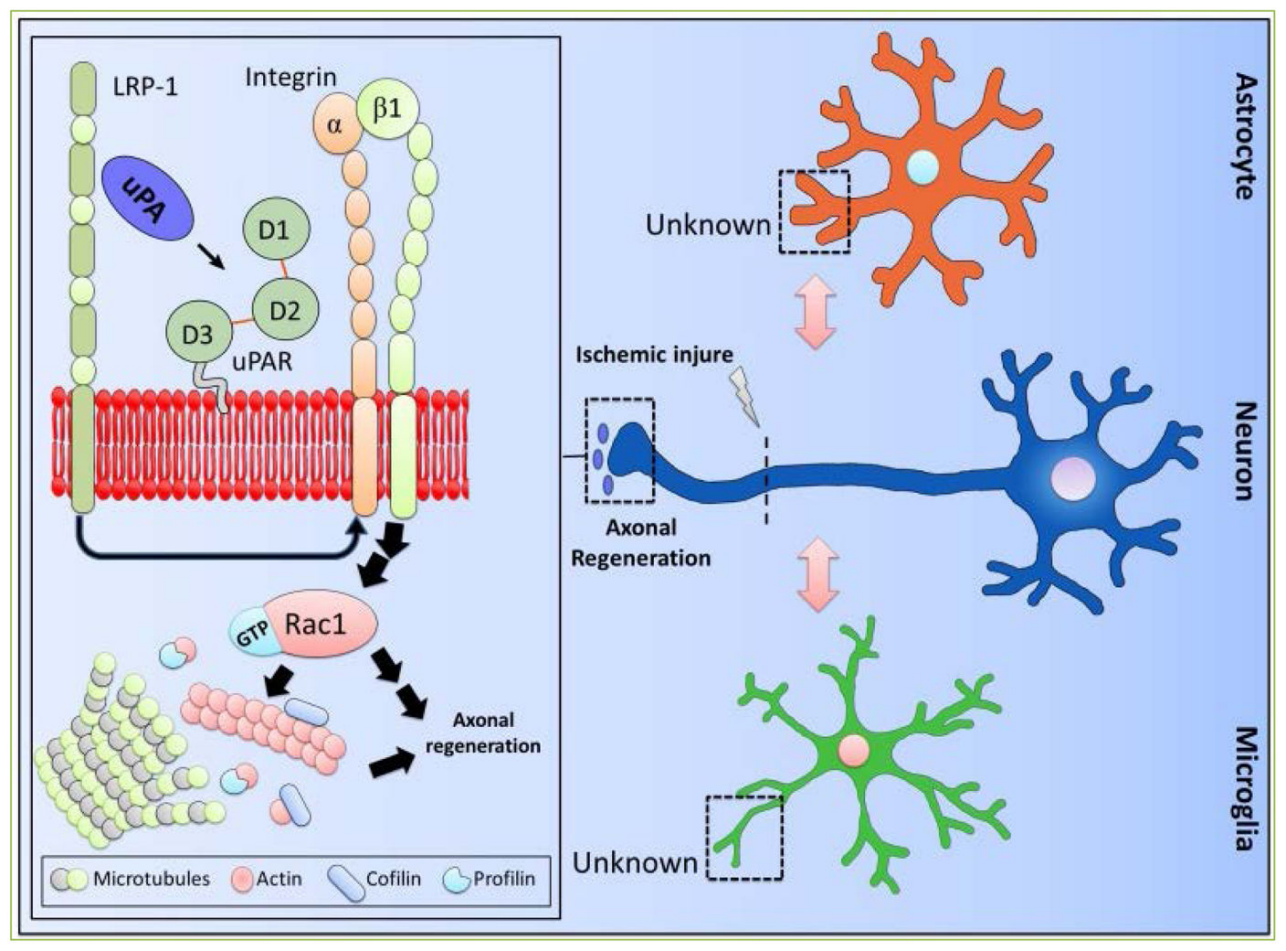
Schematic representation of the proposed model for uPA-induced axonal recovery in the ischemic brain Axonal injury causes the release of uPA and an increase in the expression of uPAR and LRP1 in the peripheral zone of the growth cones. The binding of uPA to uPAR leads to axonal regeneration via LRP1-mediated recruitment of β1-integrin to the neuronal membrane, β1-integrin-mediated Rac1 activation, and Rac1-mediated cytoskeleton reorganization. Importantly, the role of uPAR in microglia and astrocytes is still unclear.

Based on the data discussed above we propose a model (Figure 2) in which the interaction between uPA and uPAR in the growth cone of injured axons activates an intracellular signaling pathway that promotes axonal regeneration. More specifically, the axonal injury causes the release of uPA and an increase in the expression of uPAR and LRP1 in the peripheral zone of the growth cones. The binding of uPA to uPAR leads to LRP1-mediated recruitment of β1-integrin to the neuronal membrane, β1-integrin-mediated Rac1 activation, and Rac1-induced axonal regeneration. These observations indicate that the uPA-uPAR-LRP1 system is a potential target for the development of therapeutic strategies to promote axonal recovery in the central nervous system.
